# High-Intensity Interval Training and Moderate-Intensity Continuous Training Attenuate Oxidative Damage and Promote Myokine Response in the Skeletal Muscle of ApoE KO Mice on High-Fat Diet

**DOI:** 10.3390/antiox10070992

**Published:** 2021-06-22

**Authors:** Linjia Wang, Jessica Lavier, Weicheng Hua, Yangwenjie Wang, Lijing Gong, Hao Wei, Jianxiong Wang, Maxime Pellegrin, Grégoire P. Millet, Ying Zhang

**Affiliations:** 1 School of Sport Science, Beijing Sport University, Beijing 100084, China; 1004320180046@bsu.edu.cn (L.W.); 2019210254@bsu.edu.cn (W.H.); youngn@bsu.edu.cn (Y.W.); 2020110074@bsu.edu.cn (H.W.); 2 Institute of Sport Sciences, University of Lausanne, 1015 Lausanne, Switzerland; jessica.lavier@unil.ch; 3 Division of Angiology, Heart and Vessel Department, Lausanne University Hospital (CHUV), 1011 Lausanne, Switzerland; Maxime.Pellegrin@chuv.ch; 4 China Institute of Sport and Health Science, Beijing Sport University, Beijing 100084, China; lijing.gong@bsu.edu.cn; 5 Faculty of Health, Engineering, and Sciences, University of Southern Queensland, Toowoomba, QLD 4350, Australia; wangj@usq.edu.au

**Keywords:** HIIT, atherosclerosis, ApoE KO mice, skeletal muscle, oxidative damage, myokine production

## Abstract

The purpose of this study was to investigate the effects of high-intensity interval training (HIIT) and moderate-intensity continuous training (MICT) on the skeletal muscle in Apolipoprotein E knockout (ApoE KO) and wild-type (WT) C57BL/6J mice. ApoE KO mice fed with a high-fat diet were randomly allocated into: Control group without exercise (ApoE^−/−^ CON), HIIT group (ApoE^−/−^ HIIT), and MICT group (ApoE^−/−^ MICT). Exercise endurance, blood lipid profile, muscle antioxidative capacity, and myokine production were measured after six weeks of interventions. ApoE^−/−^ CON mice exhibited hyperlipidemia and increased oxidative stress, compared to the WT mice. HIIT and MICT reduced blood lipid levels, ROS production, and protein carbonyl content in the skeletal muscle, while it enhanced the GSH generation and potently promoted mRNA expression of genes involved in the production of irisin and BAIBA. Moreover, ApoE^−/−^ HIIT mice had significantly lower plasma HDL-C content, mRNA expression of *MyHC-IIx* and *Vegfa165* in EDL, and ROS level; but remarkably higher mRNA expression of *Hadha* in the skeletal muscle than those of ApoE^−/−^ MICT mice. These results demonstrated that both exercise programs were effective for the ApoE KO mice by attenuating the oxidative damage and promoting the myokines response and production. In particular, HIIT was more beneficial to reduce the ROS level in the skeletal muscle.

## 1. Introduction

Atherosclerosis can pathologically affect the large arteries of the heart, as well as the peripheral arteries in the human body [1,2]. In the peripheral arteries, atherosclerosis has a considerable effect on skeletal muscle structure and function. It has been reported that individuals with peripheral arterial disease (PAD) usually suffer a myopathy in the diseased limbs caused by the oxidative damage and mitochondrial disorder [3,4]. Apolipoprotein E (ApoE) is an important component of all plasma lipoproteins and serves as a ligand for the cell-surface lipoprotein receptors, such as the LDL-receptor. ApoE knockout (KO) mice spontaneously develop hypercholesterolemia and atherosclerosis when fed standard chow [5]. It has also been observed that a high-fat diet (HFD) can further exacerbate and accelerate these lesions [6]. Thus, ApoE KO is a commonly used animal model in atherosclerosis research.

Physical activity has been demonstrated to be a preventive tool for the development of atherosclerosis [7,8]; however, most current research has investigated the effects of exercise on the severity and composition of atherosclerotic plaque in cardiovascular diseases, whereas the impacts on the skeletal muscle are not well studied. Specifically focused on the ApoE KO mice with PAD, our team has found that neither running on the treadmill or wheel nor swimming enhanced vascularization and blood flow in the ischemic limb, as well as negligible changes in glucose metabolism and mitochondrial biogenesis markers in the skeletal muscles [9]. These results suggest that not all kinds of exercise are beneficial to the ApoE KO mice. Therefore, investigating the most effective exercise modality with different intensities and durations is worthy of interest.

The high-intensity interval training (HIIT) consists of brief intervals of vigorous activity interspersed with periods of low activity or rest and has been employed to improve athletic exercise performance since the mid-20th century. Contemporary protocols developed for non-athletes are intended to reduce exercise time and provide a greater stimulus for physiological adaptation, compared with moderate-intensity continuous training (MICT) [10,11]. Recent studies have shown an improvement in both whole-body and skeletal muscle metabolic health in different populations following HIIT [12]. In particular, HIIT has been touted as the most beneficial exercise intervention for patients with cardiovascular disease [13]. However, the effects of HIIT intervention on the skeletal muscle of ApoE KO mice on HFD, compared with MICT, have not been studied to date.

Oxidative stress is an important manifestation of peripheral atherosclerosis in the skeletal muscle [6,14]. At higher concentrations, reactive oxygen species (ROS) may lead to oxidative stress and oxidative damage of biomolecules [15]. The increased ROS production by dysfunctional mitochondria in the leg muscles of PAD patients has been recognized as the key mechanism of initiation and progression of the disease [3,16]. Previous studies have also observed elevated levels of 4-hydroxynonenal (4-HNE) and protein carbonyls in PAD muscle specimens [17,18]. NADPH oxidases (Noxs) are ROS-generating enzymes. Skeletal muscles express three isoforms of Noxs (Nox1, Nox2, and Nox4) that have been identified as critical modulators of redox homeostasis [19]. Among the three isoforms, Nox2 acts as the main source of skeletal muscle ROS during contractions [19,20,21] and the subunit p47phox is required for Nox2 activity [22]. It is well-known that the transcription factor nuclear factor erythroid 2-related factor 2 (Nrf2) is the master regulator of cellular antioxidant defense, because it regulates more than 200 cytoprotective genes in response to oxidative stress [23]. Thus, it is important to understand the effects of HIIT and MICT on redox homeostasis and the adaptative response of Nrf2 pathway expression. In addition, skeletal muscle morphology may be transformed by hyperlipidemia. ApoE deficiency and Western-type diet independently induced a higher capillary-to-fiber ratio in the glycolytic extensor digitorum longus (EDL) followed by increased mRNA levels of key angiogenic factors [24], leading to an enhanced transition to more oxidative myofibers i.e., the transition of fast-twitch glycolytic IIb to the fast-twitch IIx fibers, in ApoE KO mice [24,25].

The skeletal muscle is the major organ contributing to the whole-body metabolism, and the identification of exercise-induced myokines set a new paradigm in exercise biology and metabolic homeostasis. In the past few decades, a great number of myokines have been discovered, and among them are irisin, FNDC5/irisin, β-aminoisobutyric acid (BAIBA), and musclin [26,27]. A lack of exercise, which is associated with a network of diseases, including hyperlipidemia, type 2 diabetes, cardiovascular diseases, and others [28,29], leads to an altered response of the secretion of myokines and/or resistance to them. Thus, myokines could serve as the prognostic biomarkers, which reflect the benefits of exercise on metabolism [26]. However, the effects of exercise modality (HIIT vs. MICT) on the production of irisin, BAIBA, and musclin in skeletal muscles of ApoE KO mice still remains poorly understood. In this context, the purpose of the present study was to investigate and compare the effects of six weeks of HIIT and MICT on the hypercholesterolemia model of ApoE KO mice fed HFD, with a special focus on the changes in redox homeostasis and myokine production in the skeletal muscle. We hypothesized that the two training modalities would present remarkable impacts on the measured variables, with HIIT being superior to MICT for improving the pro-/antioxidant status of the skeletal muscle.

## 2. Materials and Methods

### 2.1. Animals

The protocols of this study were approved by the Animal Care and Use Committee of Beijing Sport University. Male wild-type (WT) C57BL/6J mice (9 weeks old, n = 10) and male C57BL/6J ApoE-KO mice (9 weeks old, n = 35) were purchased from Charles River Development, Inc. (Beijing, China), respectively. All mice were housed indoors under a temperature of 22 ± 2 °C, humidity of 50–70%, 12-h light/dark cycles and they had *ad libitum* access to deionized water and food.

Five ApoE KO mice were used to determine the maximal running speed on the treadmill and the remaining 30 ApoE KO mice were randomly allocated into three groups: ApoE KO control (ApoE^−/−^ CON), ApoE KO mice with HIIT (ApoE^−/−^ HIIT), and ApoE KO mice with MICT (ApoE^−/−^ MICT), with 10 mice in each group. All ApoE KO mice were fed the HFD, containing 21% (*w/w*) fat and 1.5% (*w*/*w*) cholesterol (Beijing Keao Xieli Feed Co., Ltd., Beijing, China), following a three-day standard chow then a three-day mixed diet of standard chow and a gradually increased proportion of HFD. In the meantime, the WT mice were fed standard chow, containing 4–5% (*w*/*w*) fat and no cholesterol (Beijing Huafukang Bioscience Co., Ltd., Beijing, China). In addition, in the ApoE^−/−^ HIIT and ApoE^−/−^ MICT groups, there was a 4-day adaptive training session before the start of the training program. The adaptive training program was conducted by running on a treadmill 10 min per day, and the speed was gradually increased every day from 10 cm/s, 12 cm/s, 14 cm/s to 16 cm/s from day 1 to day 4, respectively. Overall, the acclimatization of animals to their housing, chow, and treadmill running lasted one week.

### 2.2. Determination of the Maximal Running Speed on Treadmill

Five ApoE KO mice performed a treadmill running test, which started at 4.8 m/min for 10 min with an incline of 0° and the speed was progressively increased 1.2 m/min every 3 min until exhaustion. The exhaustion was judged when the mouse stayed still either for three seconds on the electric grid or received 100 shocks without moving [30]. The last speed was defined as the maximal running speed.

### 2.3. Training Protocols

The HIIT program was described before [30], with slight modification, which consisted of 4 sets of 5 × 10-s sprints with 20 s of rest between each sprint and the interset rest was 5 min. One training session took about 23 min in total. The exercise intensity of the sprint was about 100% of the measured maximal running speed. On the other hand, the ApoE^−/−^ MICT group performed the continuous endurance running for 40 min with a speed at 40% of the determined maximal running speed. All training sessions for the two groups were carried out in the morning, three times per week, for six weeks.

### 2.4. Assessment of Endurance Exercise Performance

The mice in the ApoE^−/−^ HIIT and ApoE^−/−^ MICT groups performed an incremental treadmill running test to exhaustion after the training intervention. The protocol was the same as the one used to determine the maximal running speed. The running distance was recorded as the endurance exercise performance. After the incremental treadmill exercise, the mice rested for at least 48 h. Then they were anesthetized and blood samples were collected by the percutaneous cardiac puncture. The muscle samples from gastrocnemius, soleus, and extensor digitorum longus (EDL) were removed, cleaned, and quick-frozen in liquid nitrogen, and then stored at −80 °C.

### 2.5. Plasma Lipid Profiles

Plasma total cholesterol (TC), triglycerides (TG), low-density lipoprotein cholesterol (LDL-C), and high-density lipoprotein cholesterol (HDL-C) were measured using the specific assay kits (A111-1-1, A110-1-1, A113-1-1, and A112-1-1, respectively, Nanjing Jiancheng Bioengineering Institute, China), according to the manufacturer’s protocols. Changes in absorbance were determined with Bio Tek Synergy H1 (Bio Tek Instruments, Inc., Winooski, VT, USA) at 510, 510, 546, and 546 nm for plasma TC, TG, LDL-C, and HDL-C assays, respectively.

### 2.6. Real-time Quantitative PCR Analysis

Total RNA was isolated from about 50 mg of crushed gastrocnemius muscle the using TRIzol reagent (TaKaRa, Japan) and about 1 μg total RNA was reverse-transcribed to cDNA using a kit (FSQ-101; Toyobo Co., Ltd., Japan) according to the manufacturer’s instructions. Besides, total RNA was isolated from about 10 mg of soleus and EDL, respectively, using the RNA Isolation Kit by TransGen Biotech (Beijing, China) and about 1 μg of total RNA was reverse-transcribed to cDNA using the same kit (FSQ-101; Toyobo Co., Ltd., Japan). Moreover, the real-time qPCR was performed in an ABI 7500 Real-time PCR System (Thermo Scientific, Inc., Waltham, MA, USA) using the SYBR Green Real-time PCR Master Mix kit (Toyobo Co., Ltd., Osaka, Japan) with the previously synthesized cDNA as a template in a 20 µL reaction volume. Glutamate-cysteine ligase catalytic subunit (*Gclc*; gene ID: 14629; QT00130543), glutathione reductase (*Gsr*; gene ID: 14782; QT01758232), and 18S ribosomal RNA (*Rn18s*; gene ID: 19791; QT02448075) commercial primers from Qiagen (Germany) were used.

In addition, the primer sequences of glutamate-cysteine ligase modifier subunit (*Gclm*), glutathione synthase (*Gss*), NADPH oxidase 2 (*Nox2*) (also called *gp91phox*), neutrophil cytosolic factor 1 (*Ncf1*) (also called *p47phox*), NADPH oxidase 4 (*Nox4*), fibronectin type III domain containing 5 (*Fndc5*), acyl-CoA dehydrogenase short chain (*Acads*), hydroxyacyl-CoA dehydrogenase trifunctional multienzyme complex subunit alpha (*Hadha*), and hydroxyacyl-coenzyme A dehydrogenase *(Hadh)* were listed in Table 1 and these primers were synthesized by Invitrogen Trading Co., Ltd. (Shanghai, China). The difference in expression between control and experimental samples was calculated using the 2^−^^△△Ct^ method, as described previously [31].

The measurement samples for mRNA expression of vascular endothelial growth factor a 165 (*Vegfa165*) were from soleus and EDL, respectively; the samples for mRNA expression of myosin heavy chain *(MHC)-IIa, IIx, and IIb* were from EDL. Their primers were also synthesized by Invitrogen Trading Co., Ltd. (Shanghai, China) and were listed in Table 1.

### 2.7. Western Blotting

Total proteins were isolated from 50 mg of gastrocnemius using RIPA protein extraction reagents (P0013B; Beyotime, Inc., Shanghai, China). Protein concentration was measured using the BCA protein assay kit (Pierce 23225; Thermo Fisher Scientific, Inc.). Twenty micrograms of proteins were separated on Bolt 4–12% Bis-Tris PlusGels (NW04125BOX; Thermo Fisher Scientific, Inc., Waltham, MA, USA.) by electrophoresis, and the fractionated proteins were subsequently transferred to a nitrocellulose membrane using iBlot Gel Transfer Stacks Nitrocellulose (IB23001; Thermo Fisher Scientific, Inc.). The blots were probed using the following antibodies: Nuclear factor erythroid-derived 2-like 2 (Nrf2) (1:200, sc-365949; Santa Cruz Biotechnology, Dallas, TX, USA), Ser40-phosphorylated (p)-Nrf2 (P-Nrf2) (1:200, bs-2013R; Bioss, Beijing, China), 4-HNE-modified protein (1:500, ab-46545; Abcam, Cambridge, MA, USA), catalase (CAT) (1:10,000, 66765-1-Ig, Proteintech, Huhan, China), superoxide dismutase 1 (SOD1) (1:500, sc-11407; Santa Cruz Biotechnology, USA), NAD (P)H quinone oxidoreductase 1 (NQO1) (1:500, sc-32793; Santa Cruz Biotechnology, USA), glutathione peroxidase 1 (GPX1) (1:500, ab-108427; Abcam, USA), and β-actin (1:1000, sc-47778, Santa Cruz Biotechnology, Dallas, TX, USA). The density of protein bands was analyzed using Bio-Rad imaging software (Bio-Rad Laboratories, Hercules, CA, USA). The individual values were originally expressed as a ratio of a standard (β-actin content) and then expressed as a fold change of the control group value.

### 2.8. Reactive Oxygen Species (ROS) Generation

Following the manufacturer’s instructions, 50 mg of gastrocnemius muscles were homogenized with reagent C in the kit (GMS10016.3; GENMED, Shanghai, China). The supernatants were used to yield ROS samples (2 g protein/L). Then ROS samples were incubated with the chloromethyl derivative (CM-H2DCFDA) of 5-(and-6)-chloromethyl-2′,7′-dichlorodihydrofluorescein diacetate (H2DCFDA) at 37 °C for 20 min in the 96-well plates, and the ROS levels were detected by a fluorescence plate reader at λexc: 490 nm and λem: 520 nm (Bio Tek Synergy H1, Bio Tek Instruments) as previously described [32].

### 2.9. Glutathione Redox State and Protein Carbonyl Content of the Skeletal Muscle

Glutathione redox state (the ratio of reduced glutathione (GSH) to oxidized glutathione (GSSG); GSH/GSSG) was measured from 50 mg of the gastrocnemius muscle by GSH and GSSG commercial kits from Solarbio (BC1175 and BC1185, Beijing, China), according to the manufacturer’s protocols. Protein carbonyl content was assayed in the homogenate supernatant of 50 mg of gastrocnemius tissue using the commercial assay kit purchased from Solarbio (BC1275, Beijing, China), according to the manufacturer’s instructions.

### 2.10. Plasma Irisin and Muscle Musclin Concentration

Plasma irisin and musclin concentration in the gastrocnemius were assessed according to the manufacturer’s instructions with the mouse irisin and musclin ELISA kits (Gene lab., Beijing, China), respectively. The plates were read at 450 nm (Bio Tek Synergy H1, Bio Tek Instruments, Inc., Winooski, VT, USA).

### 2.11. Statistical Analysis

All values were presented as the mean ± standard error (SE). Statistical analyses were performed using SPSS Statistical software V 19.0 (IBMCorp., Armonk, NY, USA). Comparisons between the means of the WT mice and ApoE^−/−^ CON groups were made using the independent sample *t*-test. The one-way ANOVA was used to analyze the impact of different interventions on the ApoE^−/−^ mice followed by the least significant difference (LSD) post hoc test at *p* < 0.05 level of significance.

## 3. Results

### 3.1. Body Weight, Running Distance, and Plasma Lipid Profiles

**Figure 1:**
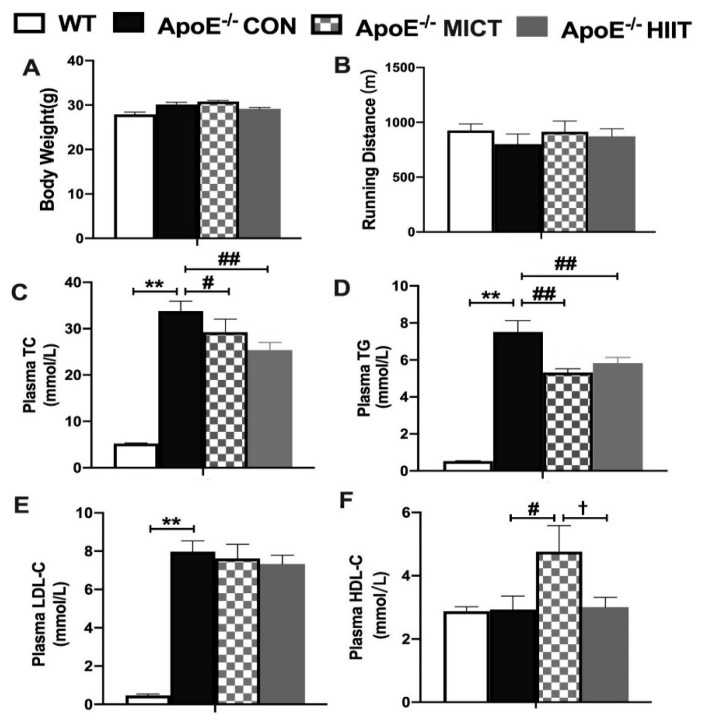
Effects of HIIT and MICT on body weight (**A**), running distance (**B**), and plasma lipid profiles (**C**–**F**) in WT mice and ApoE KO mice on HFD. Values are displayed as the mean ± SEM (n = 10/group). ** *p* < 0.01 vs. WT; # *p* < 0.05 vs. ApoE^−/^^−^ CON; ## *p* < 0.01 vs. ApoE^−/^^−^ CON; † *p* < 0.05 vs. ApoE^−/^^−^ MICT.

There were no significant differences in body weight and running distance between the ApoE^−/−^ CON group and WT mice and among CON, MICT, and HIIT groups of ApoE^−/−^ mice (Figure 1A,B). However, significantly higher levels of plasma TC, TG, and LDL-C were observed in the ApoE^−/−^ CON group than those of the WT group (Figure 1C–E). Meanwhile, the ApoE^−/−^ HIIT and ApoE^−/−^ MICT groups had significantly lower plasma TC and TG levels, while the ApoE^−/−^ MICT group had a significantly higher plasma HDL-C level than those of the ApoE^−/−^ CON group (Figure 1C,D). In addition, the ApoE^−/−^ MICT group had a significantly higher plasma HDL-C level than that of the ApoE^−/−^ CON and ApoE^−/−^ HIIT groups, respectively (Figure 1F).

### 3.2. The mRNA Expression of Vegfa165, MyHC-IIa, MyHC-IIx, and MyHC-IIb in Soleus or EDL

**Figure 2:**
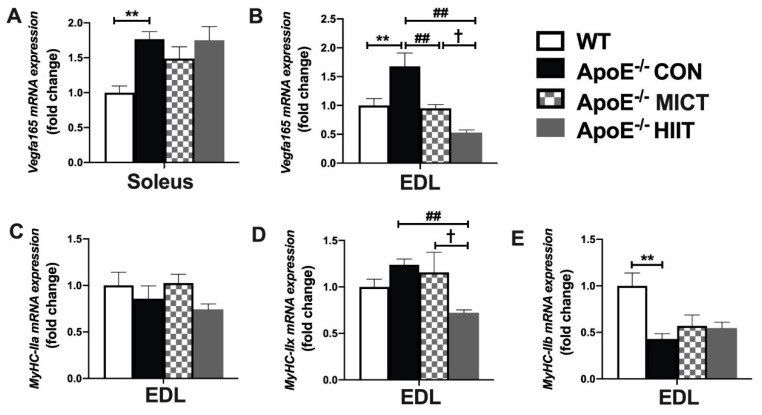
Effects of HIIT and MICT on the mRNA expression of *Vegfa165* in soleus (**A**) and EDL (**B**), and *MyHC-IIa*, *MyHC-IIx*, and *MyHC-IIb* in EDL (**C**–**E**) of WT and ApoE KO mice on HFD. Values are displayed as the mean ± SEM (n = 10/group). ** *p* < 0.01 vs. WT; ## *p* < 0.01 vs. ApoE^−/^^−^ CON; † *p* < 0.05 vs. ApoE^−/^^−^ MICT.

The mRNA expression of *Vegfa165* in soleus and EDL was significantly higher, and *MyHC-IIb* in EDL was significantly lower in the ApoE^−/−^ CON group, compared to those of WT mice (Figure 2A,B,E). However, six weeks of the HIIT and MICT induced a significantly lower mRNA expression of *Vegfa165* in EDL, respectively, and the HIIT group had a markedly lower mRNA expressions of *MyHC-IIx* in EDL, compared with those of the Apo KO control mice (Figure 2B,D). Moreover, the mRNA expression of *Vegfa165* and *MyHC-IIx* in EDL of ApoE^−/−^ HIIT mice was significantly lower than those of ApoE^−/−^ MICT mice (Figure 2B,D). There were no significant differences in *MyHC-IIxa* between the ApoE^−/−^ CON group and WT mice and among CON, MICT, and HIIT groups of ApoE^−/−^ mice (Figure 2C).

### 3.3. Muscle ROS, Protein Carbonyl, 4-HNE Modified Proteins, and the mRNA Expression Levels of Nox2, p47phox and Nox4

**Figure 3:**
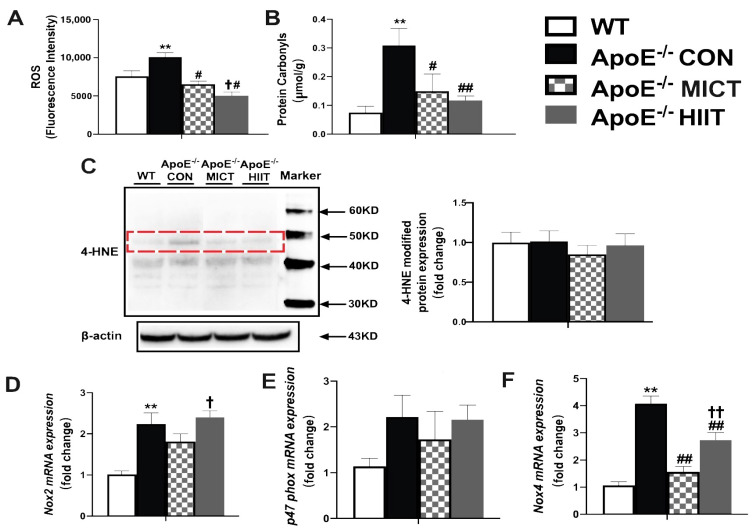
Effects of HIIT and MICT on ROS generation (**A**), protein carbonyl (**B**), 4-HNE-modified proteins (**C**), and the mRNA expression levels of *Nox2*, *P47phox*, and *Nox4* (**D**–**F**) in skeletal muscles of WT and ApoE KO mice with HFD. Values are displayed as the mean ± SEM (n = 10/group). ** *p* < 0.01 vs. WT; # *p* < 0.05 vs. ApoE^−/^^−^ CON; ## *p* < 0.01 vs. ApoE^−/^^−^ CON; † *p* < 0.05vs. ApoE^−/^^−^ MICT; †† *p* < 0.01 vs. ApoE^−/^^−^ MICT.

There were higher levels of ROS and protein carbonyl and mRNA expression of *Nox2* and *Nox4* in skeletal muscles of ApoE^−/−^ CON mice than those of WT mice (Figure 3A,B,D,F); while the expressions of 4-HNE protein and p47 phox mRNA were not different between the ApoE^−/−^ CON group and WT mice and among CON, MICT, and HIIT groups of ApoE^−/−^ mice (Figure 3C,E). Moreover, the treatments of HIIT and MICT resulted in significantly lower levels of the muscle ROS, protein carbonyl, and mRNA expression of *Nox4*, compared with those of the ApoE^−/−^ CON mice (Figure 3A,B,F). In addition, ROS level was significantly lower and the mRNA expression of *Nox2* and *Nox4* were significantly higher in gastrocnemius muscles of ApoE^−/−^ HIIT mice, compared with those of ApoE^−/−^ MICT mice (Figure 3A,D,F).

### 3.4. The mRNA Expression of Genes Involved in the Production of GSH, GSH, GSSG Levels, and GSH/GSSG Ratio

**Figure 4:**
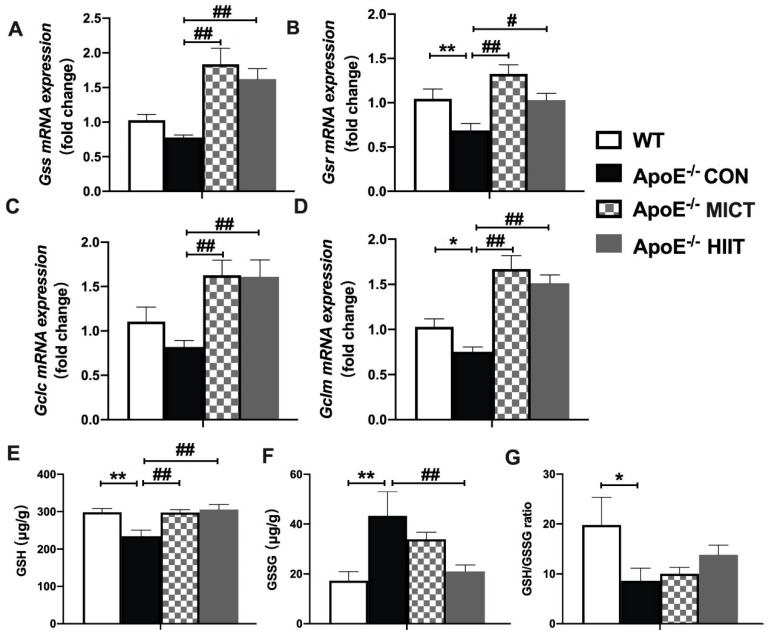
Effects of HIIT and MICT on the mRNA expression of genes involved in the production of GSH (**A**–**D**), GSH (**E**), GSSG (**F**) levels, and GSH/GSSG ratio (**G**) in skeletal muscles of WT and ApoE KO mice with a high-fat diet. Values are displayed as the mean ± SEM (n = 10/group). * *p* < 0.05 vs. WT; ** *p* < 0.01 vs. WT; # *p* < 0.05 vs. ApoE^−/^^−^ CON; ## *p* < 0.01 vs. ApoE^−/^^−^ CON.

The mRNA expression of genes (*Gsr and Gclm*) involved in the production of GSH was significantly lower in the skeletal muscle of ApoE^−/−^ CON mice, compared to those of the WT mice (Figure 4B,D). Moreover, the treatment of HIIT and MICT produced significantly higher expression in all measured genes involved in the production of GSH (*Gss*, *Gsr*, *Gclc*, and *Gclm*) in the skeletal muscle, compared with those of the Apo KO control mice (Figure 4A–D).

The lower level of GSH and the higher level of GSSH resulted in a reduced GSH/GSSG ratio in the ApoE^−/−^ CON group, compared to those of the WT mice (Figure 4E–G). Moreover, the HIIT and MICT treatments did not exhibit a significant difference in the GSH/GSSG ratio, compared with that of the ApoE^−/−^ CON group, although the GSH levels were increased in the ApoE^−/−^ HIIT and ApoE^−/−^ MICT groups, and GSSG level was decreased in the ApoE^−/−^ HIIT group (Figure 4E–G). In addition, there was no significant difference in these measured indexes between ApoE^−/−^ HIIT and ApoE^−/−^ MICT mice (Figure 4A–G).

### 3.5. The Protein Expression of Nrf2, p-Nrf2 (ser40), and Antioxidants

**Figure 5:**
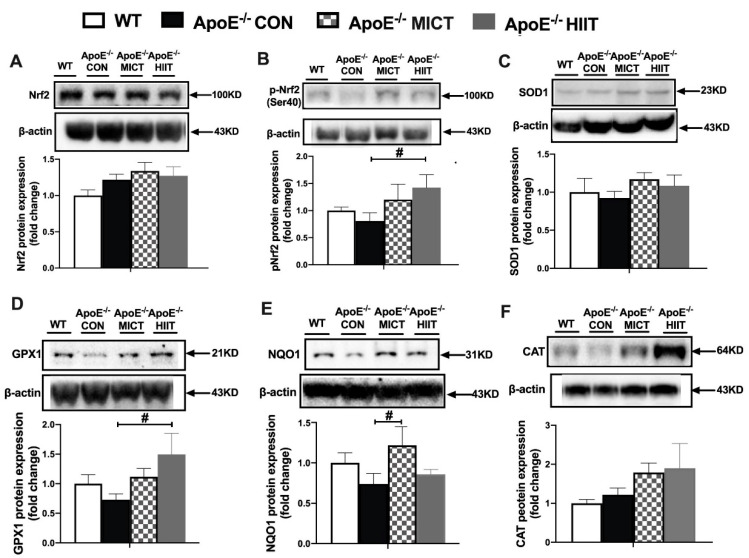
Effects of HIIT and MICT on the protein expression of Nrf2 (**A**), p-Nrf2 (ser40) (**B**), and antioxidants (**C**–**F**) in skeletal muscles of WT and ApoE KO mice with HFD. Values are displayed as the mean ± SEM (n = 10/group). # *p* < 0.05 vs. ApoE^−/^^−^ CON.

There were no significant differences in the protein expression of Nrf2, p-Nrf2 (ser40), and all measured antioxidants between ApoE^−/−^ CON and WT mice (Figure 5A–F). Moreover, there was significantly higher protein expression of p-Nrf2 (ser40) and GPX1 in ApoE^−/−^ HIIT mice, and a prominently higher protein expression of NQO1 in ApoE^−/−^ MICT mice, compared with ApoE^−/−^ CON mice, respectively (Figure 5B,D,E). There was no significant difference in these measured protein expression levels between ApoE^−/−^ HIIT and ApoE^−/−^ MICT (Figure 5A–F).

### 3.6. The mRNA Expression of Fndc5, Hadh, Acads, and Hadha in Gastrocnemius, Plasma Irisin Level, as Well as Muscle Musclin Concentration

**Figure 6:**
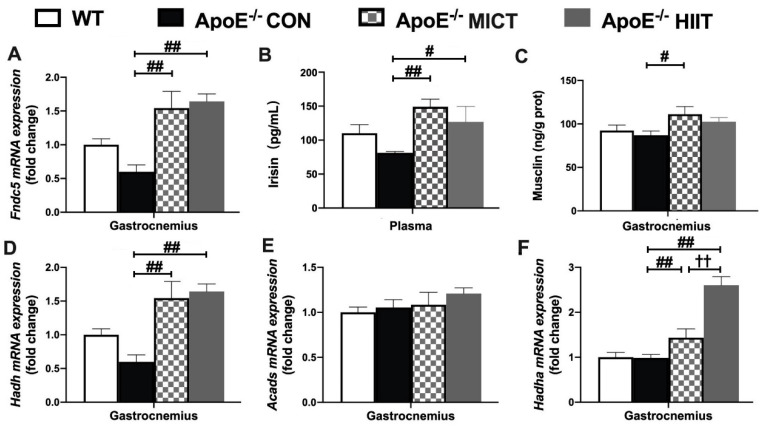
Effects of HIIT and MICT on the mRNA expression of Fndc5 (**A**), Hadh, Acads, and Hadha (**D**–**F**), and musclin level (**C**) in the gastrocnemius as well as plasma irisin level (**B**) of WT and ApoE KO mice with HFD. Values are displayed as the mean ± SEM (n = 10/group). # *p* < 0.05 vs. ApoE^−/^^−^ CON; ## *p* < 0.01 vs. ApoE^−/^^−^ CON; †† *p* < 0.01 vs. ApoE^−/^^−^ MICT.

There was no significant difference in the mRNA expression of *Fndc5*, *Hadh, Acads*, and *Hadha*, and musclin content in the gastrocnemius, as well as plasma irisin level between the WT and ApoE^−/−^ CON groups (Figure 6A–F). However, significantly higher mRNA expression of *Fndc5* (Figure 6A), *Hadh*, and *Hadha* in the gastrocnemius (Figure 6D,F), as well as plasma irisin level (Figure 6B), were observed in the ApoE^−/−^ MICT and ApoE^−/−^ HIIT groups than those of the ApoE^−/−^ CON group. Meanwhile, there was a significantly higher musclin level of the gastrocnemius of ApoE^−/−^ MICT than that of the ApoE^−/−^ CON group (Figure 6C). In addition, there was a significantly higher mRNA expression of *Hadha* in the gastrocnemius of ApoE^−/−^ HIIT mice than that of ApoE^−/−^ MICT mice (Figure 6F).

## 4. Discussion

The main findings revealed that concurrent 6-week HIIT and MICT protocols improved blood lipid profiles, counteracted ROS production and protein carbonylation in the gastrocnemius muscle, and decreased the mRNA level of the angiogenic gene *Vegfa165* in the EDL muscle. At the same time, both HIIT and MICT enhanced the GSH generation and potently promoted mRNA expression of genes involved in the production of irisin and BAIBA in the gastrocnemius muscle of ApoE KO mice. Comparison of the two training outcomes indicated that HIIT was more efficient than MICT in decreasing the ROS level of the skeletal muscle, whereas MICT was more efficient in increasing the plasma HDL-C level. To our knowledge, this is the first study to report and compare that HIIT and MICT, as potential adjuvant treatments, can attenuate oxidative damage and promote the myokine response in skeletal muscles of ApoE KO mice on HFD. These results support the hypothesis of the present study.

Our results of body weight and blood lipid profiles were in line with previous studies with ApoE KO mice on HFD exhibiting significant increases in plasma TC, TG, and LDL-C levels [33] but not becoming obese [24,34]. This may come from both lower synthesis and increased hydrolysis of triacylglycerols from the ApoE^−/−^ adipocytes [34,35]. It confirms that this mouse model is a valid model of hyperlipidemia. The present study also provided relevant evidence that ApoE KO with HFD resulted in increased mRNA expression of *Vegfa165*, and decreased mRNA expression of *MyHC-IIb*, especially in EDL. These changes in the skeletal muscle represent a functional adaptation to a hyperlipidemic environment or compensation for the excess fat. Furthermore, the ROS production and protein carbonyl in the ApoE^−/−^ CON group were higher, and the value of GSH generation was lower as compared with the WT group, confirming that hyperlipidemia increases oxidative stress in the skeletal muscle. This finding was further supported by the evidence of increased mRNA expression of Noxs genes (*Nox2*, *p47 phox*, and *Nox4*) in response to ApoE KO on HFD. The ApoE^−/−^ CON mice of the present study showed increased markers of muscular oxidative stress, the same as the mouse model of the previous studies [16,24]. However, our ApoE^−/−^ CON mice did not have a higher level of 4-HNE-modified proteins (the end products of lipid peroxidation) in the skeletal muscle, compared to that of the WT mice.

It is well-known that regular exercise has preventive effects on various organs of atherosclerosis-prone ApoE KO mice [33,36]. However, until now, there were no reports about the benefits of exercise on the skeletal muscle of ApoE KO mice on HFD. Investigating the modalities of exercise treatment (i.e., exercise duration and exercise intensity) is therefore paramount when evaluating its effects. In the present study, we applied HIIT and MICT programs and found that both of them improved plasma lipid profiles and counteracted the compensatory enhanced EDL capillarization caused by ApoE KO with HFD. Importantly, while ApoE^−/−^ CON mice impaired muscle redox homeostasis, the two training programs did attenuate the oxidative damage as shown by decreased ROS production, protein carbonyl content, and mRNA expression of *Nox4*, and also increased mRNA expression of genes involved in GSH production, GSH level, and the protein expression of some antioxidase in the skeletal muscle of these mice. This means that the two training modalities could induce adaptive responses, which were beneficial for the organism.

We found that ROS level was remarkably lower in HIIT mice than MICT mice, which implied that the magnitude of training adaptation was in part dependent upon exercise intensity, so that higher training intensities induced greater changes in the antioxidant defense [37], although there were no significant differences in measured variables of pro/antioxidant balance, including the levels of protein carbonyl, 4-HNE, GSH, and the protein expression of some antioxidase in the skeletal muscle between the two groups. Even for the Noxs, a key ROS generator during muscle contractions, the mRNA expression of *Nox2* and *Nox4* was significantly higher in ApoE^−/−^ HIIT mice than ApoE^−/−^ MICT mice. It indicated that in the skeletal muscle of HIIT mice, the antioxidant system could rapidly remove ROS before they caused cellular dysfunction and was more robust than that of the MICT group. As shown in Figure 5, HIIT mice had a significantly higher protein expression of p-Nrf2 (ser40) in the skeletal muscle, whereas MICT mice did not, compared with the ApoE^−/−^ CON group. Therefore, based on the current results, we speculated that the lower ROS level could be closely linked to the higher protein expression of p-Nrf2 (ser40) in HIIT mice. However, there might be other proteins and muscle antioxidant enzymes, other than our measured antioxidants, involved in reducing the muscle ROS level of the HIIT group. Further research is needed to address them.

Furthermore, in the present study, it was found that the mRNA expression of *MyHC-IIx* and *Vegfa165* in EDL of ApoE^−/−^ HIIT mice was significantly lower than those of ApoE^−/−^ MICT mice. This result may suggest that HIIT could have more potent effects on the resistance to the transition to slower myofibers and enhanced capillarization caused by ApoE deficiency and HFD. However, it was interesting to note that HIIT was not superior to MICT in altering blood lipids of ApoE KO mice on HFD, especially in the change of HDL-C. The change of HDL-C seems to be sensitive to training volume rather than exercise intensity.

Analyses of the skeletal muscle secretome revealed that numerous myokines are produced in response to muscle contraction, and then these factors not only regulate energy demand, but also contribute to the broad beneficial effects of exercise [27]. Myokines may be useful biomarkers for monitoring exercise prescription [38]. It has been reported that endurance exercise training upregulates peroxisome proliferator-activated receptor coactivator 1 (PGC-1) in the skeletal muscle [39,40] and the PGC-1α overexpression in the skeletal muscle increases the production of Fndc5, a precursor form of irisin, and irisin then stimulates the transformation of white adipose tissue to brown adipose tissue [41]. A prospective population-based study showed that higher serum irisin levels are associated with lower prevalence and progression of coronary atherosclerosis [42]. Protective effects of irisin on atherosclerosis were reported in two different ApoE KO mouse models [43,44]. BAIBA was also revealed to induce browning of the white adipocyte and stimulate hepatic β-oxidation. In humans, plasma BAIBA levels were increased with exercise and inversely associated with metabolic risk factors, such as fasting glucose, insulin, homeostasis model assessment of insulin resistance (HOMA-IR), and the levels of TG and TC [45]. In addition, musclin is an exercise-stimulated myokine [46] and its expression level is tightly regulated by nutritional changes, and its physiological role could be linked to glucose metabolism [47]. In the present study, we did not find significant changes in the mRNA expression of *Fndc5* and genes required for BAIBA biosynthesis (*Hadh, Acads*, and *Hadha*), musclin content in the skeletal muscle, and plasma irisin level between ApoE^−/−^ CON and WT mice. Meanwhile, the mRNA expression of *Fndc5*, *Hadh*, and *Hadha*, and musclin content in the skeletal muscle and blood irisin were upregulated in response to HIIT or MICT. Our results are in accordance with previous research that showed that muscle contraction stimulated myokine (irisin, BAIBA, and musclin) production [46,48,49]. However, it is worth noting that the mRNA expression of *Hadha*, the key gene involved in BAIBA biosynthesis, in the skeletal muscle of HIIT mice, was significantly higher than that in the MICT group. Since few studies have compared the effects of HIIT and MICT training programs on BAIBA production in muscle tissue, it was only speculated that the HIIT could be superior to MICT in BAIBA production, although BAIBA content in the skeletal muscle was not measured directly in the present study.

There are some limitations in this study. We only investigated the impacts of HIIT and MICT on ApoE KO mice with HFD for six weeks. Future studies should consider a longer duration, such as 12 weeks. We also only investigated the mRNA expression of many genes, such as *MyHC-IIa*, *MyHC-IIx*, and *MyHC-IIb*, but the immunohistochemical staining or Western blots would provide further results on the morphological changes. In addition, we focused on the changes in protein expression of antioxidant enzymes in the skeletal muscle, but we did not measure the possible changes in their activity. Further studies are also needed to determine whether antioxidant enzyme activity changes for a comprehensive evaluation of the pro-/anti-oxidant balance.

## 5. Conclusions

Six weeks of HIIT or MICT programs exerted beneficial effects on ApoE KO mice on HFD by attenuating oxidative damage and promoting myokines production in the skeletal muscle. Both training modalities could decrease plasma TC and TG levels, ROS production, and protein carbonylation in the skeletal muscle; simultaneously, they increased GSH generation and mRNA expression of genes involved in the production of irisin and BAIBA. Furthermore, HIIT was more beneficial than MICT for reducing the ROS level in the skeletal muscle.

## Figures and Tables

**Table 1:** Description of primers used for quantitative real-time PCR.

Gene Name	Gene ID	Forward Primer	Reverse Primer
*Gclm*	14630	5′-AGGAGCTTCGGGACTGTATCC-3′	5′-GGGACATGGTGCATTCCAAAA-3′
*Gss*	14854	5′-CAAAGCAGGCCATAGACAGGG-3′	5′-AAAAGCGTGAATGGGGCATAC-3′
*Gclc*	14629	5′-GGGGTGACGAGGTGGAGTA-3′	5′-GTTGGGGTTTGTCCTCTCCC-3′
*Gsr*	14782	5′-CACGGCTATGCAACATTCGC-3′	5′-GTGTGGAGCGGTAAACTTTTTC-3′
*Nox2*	13058	5′-TGAATGCCAGAGTCGGGATT-3′	5′-CGAGTCACGGCCACATACA-3′
*p47phox*	17969	5′-ACACCTTCATTCGCCATATTGC-3′	5′-TCGGTGAATTTTCTGTAGACCAC-3′
*Nox4*	50490	5′-TCCATCAAGCCAAGATTCTGAG-3′	5′-GGTTTCCAGTCATCCAGTAGAG-3′
*Fndc5*	384061	5′-TTGCCATCTCTCAGCAGAAGA-3′	5′-GGCCTGCACATGGACGATA-3′
*Acads*	11409	5′-GACTGGCGACGGTTACACA-3′	5′-GGCAAAGTCACGGCATGTC-3′
*Hadha*	97212	5′-TGCATTTGCCGCAGCTTTAC-3′	5′-GTTGGCCCAGATTTCGTTCA-3′
*Hadh*	15107	5′-TGCATTTGCCGCAGCTTTAC-3′	5′-GTTGGCCCAGATTTCGTTCA-3′
*Vegfa165*	22339	5′-TGCAGGCTGCTGTAACGATG-3′	5′-GAACAAGGCTCACAGTGATTTTCT-3′
*MHC-IIa*	17886	5′-CAGCTGCACCTTCTCGTTTG-3′	5′-CCCGAAAACGGCCATCT-3′
*MHC-IIx*	17879	5′-GGACCCACGGTCGAAGTTG-3′	5′-CCCGAAAACGGCCATCT-3′
*MHC-IIb*	77579	5′-CAATCAGGAACCTTCGGAACAC-3′	5′-GTCCTGGCCTCTGAGAGCAT-3′

## Data Availability

The data used to support the findings of this study are available from the corresponding author upon request.
